# Polyunsaturated fatty acids and child neurodevelopment among a population exposed to DDT: a cohort study

**DOI:** 10.1186/s12940-019-0456-8

**Published:** 2019-02-28

**Authors:** Ángel Mérida-Ortega, Stephen J. Rothenberg, Luisa Torres-Sánchez, Lourdes Schnaas, César Hernández-Alcaraz, Mariano E. Cebrián, Rosa María García-Hernández, Rafael Ogaz-González, Lizbeth López-Carrillo

**Affiliations:** 1 Centro de Investigación en Salud Poblacional, Instituto Nacional de Salud Pública, Av. Universidad 655, Col. Sta. María Ahuacatitlán, 62100 Cuernavaca Morelos, CP Mexico; 2 Subdirección de Investigación en Intervenciones Comunitarias, Instituto Nacional de Perinatología Isidro Espinosa de los Reyes, Ciudad de México, Mexico; 3 Departamento de Toxicología, Centro de Investigación y de Estudios Avanzados del IPN, Ciudad de México, Mexico

**Keywords:** Child neurodevelopment, DDT, Polyunsaturated fatty acids

## Abstract

**Background:**

Child neurodevelopment has been positively linked to maternal intake of polyunsaturated fatty acids (PUFAs) during pregnancy; however, it is unknown if that relationship persists among populations exposed to environmental neurotoxicants.

**Objective:**

The aim of this work was to assess whether maternal dietary intake of PUFAs during pregnancy is positively associated with child neurodevelopment, whose mothers were environmentally exposed to 1,1,1-trichloro-2,2-bis(p-chlorophenyl)ethane (DDT).

**Methods:**

A prospective cohort study with 276 mother–child pairs was performed in Mexico. Neurodevelopment was assessed by Bayley Scales II from children age 1 to 30 months. Dietary PUFAs intake was estimated by Food Frequency Questionnaire at 1st and 3rd trimester of pregnancy. DDE (1,1-dichloro-2,2-bis(p-chlorophenyl) ethylene, the main metabolite of DDT) maternal serum levels were determined by electron capture gas chromatography. Longitudinal multivariate linear mixed-effects analysis, which combines mental (MDI) and motor (PDI) Bayley scales in a single model, were performed.

**Results:**

Our results show that in a sample environmentally exposed to DDT, maternal ingestion of DPA during the first trimester of pregnancy was positively associated with MDI (β = 0.10, 95% CI 0.02, 0.18) in children from 1 to 30 months. Likewise, our results suggest that dietary ALA may be also related to MDI.

**Conclusion:**

DPA may benefit neurodevelopment even in populations exposed to DDT. Our results strengthen the importance of PUFAs intake during the prenatal period.

**Electronic supplementary material:**

The online version of this article (10.1186/s12940-019-0456-8) contains supplementary material, which is available to authorized users.

## Background

Polyunsaturated fatty acids (PUFAs) constitute up to 35% of total lipids in the brain. The fetus receives PUFAs from the mother’s diet through placental diffusion whereas breastfeeding is the main source during postnatal life [1, 2]. The most common omega-3 (ω-3) and omega-6 (ω-6) dietary PUFAs are α-linolenic (ALA) and linoleic (LA) acids. The main metabolites of ALA are eicosapentaenoic (EPA), docosapentaenoic (DPA), and docosahexaenoic (DHA) fatty acids whereas the main metabolite of LA is arachidonic acid (ARA). Fish and some vegetable oils are the main dietary sources of ω-3; meanwhile vegetables, meat, eggs, corn and safflower oils are important sources of ω-6 [3, 4].

Deficiency of PUFAs may alter cell signaling and may result in behavior, learning and cognition impairments [2]. Evidence from randomized clinical trials has been suggestive but inconclusive regarding the benefit of maternal PUFAs supplementation during pregnancy on infant neurodevelopment [5]. Neurodevelopment is a complex process involving not only dietary but also genetic and environmental factors [6]. In a cohort, study including children from 9 to 30 months of age, it was found that maternal serum PUFAs status during pregnancy was positively associated with motor development, which became stronger after adjusting for prenatal methylmercury, a known neurotoxicant [7]. Maternal intake of PUFAs may improve child neurodevelopment even in populations exposed to environmental contaminants; however, there are few studies that evaluate nutrition in mothers exposed to chemical pollutants during pregnancy.

We previously reported that maternal environmental exposure to 1,1,1-trichloro-2,2-bis(p-chlorophenyl)ethane (DDT) during the first trimester of pregnancy was significantly negatively associated with motor development in children from 1 to 12 months [8]. The first trimester of pregnancy is critical for the central nervous system and neuron development [9]. DDT is a highly persistent lipophilic compound [10] that was widely used until the end of last century in Mexico [11]. It may be possible that other persistent organic pollutants have a neurotoxic effect similar to that of DDT [12].

In this report, we are evaluating if maternal intake of PUFAs is associated with child neurodevelopment in a sample environmentally exposed to DDT.

## Materials and methods

Between January 2001 and June 2009, a prospective cohort study was conducted to evaluate the association between prenatal maternal DDE serum levels and child neurodevelopment. Detailed information about cohort assembling and follow-up has been published elsewhere [8, 13].

Briefly, 1585 women were identified during prenuptial talks required to perform civil marriage in four municipalities of Morelos state, Mexico, where DDT was used until 1998 to combat endemic malaria. Eligible women had no history of chronic diseases and were not being treated with anticonvulsants. Women who refused to participate at the beginning of the study or those who were enrolled and then decided not to continue, were interviewed about their age, educational level, and occupation as well as the reason for not participating. Those women who agreed to participate (*n* = 996) and signed an informed consent letter were contacted every 8 weeks to detect pregnancies during the first trimester. They were personally interviewed about their sociodemographic, reproductive, and dietary characteristics. Women that became pregnant (*n* = 517) were followed up to assess their pregnancy evolution in each trimester. At the end of their pregnancy, 442 women remained in the cohort (75 follow-up losses = 14.5%).

Of 442 births, 41 newborns were not eligible according to one or more of the following criteria: prematurity (≤ 37 weeks), low birth weight (≤ 2 kg), twin birth, cerebral atrophy, birth defects, or perinatal asphyxia. Children were enrolled in the cohort from 2001 to 2006 and followed up until 2009. We assessed neurodevelopment at 1, 3, 6, 12, 18, 24, 30, 42, 48, 54, and 60 months of age.

**Fig. 1:**
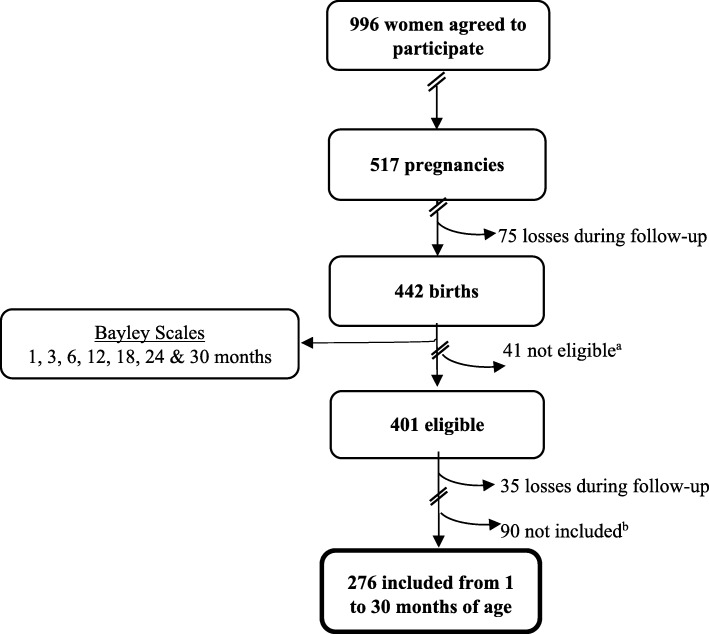
Study population selection pathway. ^a^ Prematurity (≤ 37 weeks), low birth weight (≤ 2 kg), twin birth, cerebral atrophy, birth defects, or perinatal asphyxia. ^b^ Less than two Bayley evaluations, no dietary information during first and/or third trimester of pregnancy, maternal energy intake < 500 Kcal/day and no maternal serum DDE evaluation during the first trimester of pregnancy

At 30 months of age, 366 children remained in the cohort. For the present analysis, we included 276 who had the following inclusion criteria: maternal serum DDE at first trimester of pregnancy, at least one evaluation of maternal diet during the first or third trimester of pregnancy, maternal energy intake > = 500 Kcal/day, and at least two evaluations of child neurodevelopment between 1 to 30 months of age (Fig. 1).

### Child neurodevelopment

The Spanish version of Bayley Scales of Infant Development (BSID-II) [14] is a test for children between 1 and 42 months that has been used in Mexico to evaluate mental (MDI) and psychomotor development (PDI) [15]. Two trained psychologists applied the BSID-II with an interobserver concordance of 0.96 for MDI and 0.98 for PDI. We analyzed neurodevelopment from 1 to 30 months.

### Dietary intake of ω-3 and ω-6 fatty acids

We assessed daily dietary intakes of ALA, EPA, DPA, DHA, LA, and ARA fatty acids with a validated semi-quantitative food frequency questionnaire [16], which was administered during the first and third trimesters of pregnancy. The instrument consists of 92 predetermined food portions (i.e., a glass of milk, a cup of yogurt, a spoon of oil, a slice of cheese, a plate of legumes, a piece of apple, etc.) and 7 typical dishes with consumption options from never to 6 times per day.

For each food, we obtained the content of fatty acids and other nutrients of interest (folate, vitamin B12, choline, iron, zinc) from the United States Department of Agriculture nutritional food composition reference table No. 20 [17]. Nutrient intake was adjusted using the daily energy residuals method [18].

### Serum DDE

Maternal DDE serum levels were determined during pregnancy by gas chromatography with an electron capture detector. Results were reported on lipid basis (ng/g): mean (min-max) first trimester = 2117.19 (69.44–28,410.42); second trimester = 1410.92 (52.10–8991.00); third trimester = 1376.11 (40.00–11,481.59)]. Detailed procedures, including quality control, have been previously described [8, 13].

### Maternal intelligence quotient

The maternal intellectual coefficient (IQ) was measured using a Spanish version of Wechsler Adult Intelligence Scale [19].

### Quality of stimulation at home

We evaluated parent–child communication and interaction, as well as child stimulation at home at 6 months of age using the Home Observation for Measurement of the Environment (HOME) test, which has a maximum score of 45 [20].

### Statistical analysis

Selected characteristics of participants and non-participants (exclusions plus losses to follow-up (*n* = 125)) were compared using chi-squared, Wilcoxon and T-test. We compared daily median dietary intake of PUFAs and total energy at first and third trimester. In addition, we evaluated spearman correlations between intake of PUFAs and DDE serum levels.

We evaluated the association of each PUFAs intake and child neurodevelopment with multivariate linear mixed-effects regression models [21]. We used a longitudinal data set structured where each line represents a child at a given age and for a given Bailey scale. This analysis combines motor and mental indexes of Bayley scale in a single model; therefore, there are two βs within the interaction term which correspond to the marginal effects of MDI and PDI, respectively. Final models were stated as follows:

$$ {\mathrm{Y}}_{\mathrm{i}\mathrm{j}}={\upbeta}_{\mathrm{o}+}{\mathrm{Bayley}\ \mathrm{Scales}}_{\mathrm{i}}\upbeta\ \left({\mathrm{PUFA}}_{\mathrm{i}}{\upbeta}_1+{\mathrm{X}}_{\mathrm{i}}{\upbeta}_2+{\mathrm{X}}_{\mathrm{i}}{\upbeta}_3+{\mathrm{X}}_{\mathrm{i}}{\upbeta}_4+{\mathrm{X}}_{\mathrm{i}}{\upbeta}_5+{\mathrm{X}}_{\mathrm{i}}{\upbeta}_6+{\mathrm{X}}_{\mathrm{i}}{\upbeta}_7+{\mathrm{X}}_{\mathrm{i}}{\upbeta}_8\right)+{\mathrm{Z}}_{\mathrm{i}\mathrm{j}}{\upgamma}_{\mathrm{i}}+{\upvarepsilon}_{\mathrm{i}\mathrm{j}} $$

where Y_ij_ corresponds to the PDI or MDI Bayley scores over all ages in participant _i_ and time _j_ (where _j_ = 1, 3, 6, 12, 18, 24, 30 months of age), the multiplicative interaction term included the indicator variable Bayley Scales_i_ which identify MDI as 0 and PDI as 1; and PUFA_i_ which is the intake of each PUFA during the first or third trimester of pregnancy (i.e. when the interaction term takes the value 0, it represents the marginal effect on MDI due to each PUFA intake. Likewise, the value 1 corresponds to the marginal effect on PDI). The covariables included in the model were: log base 2 DDE during the first trimester (X_i_β_2_), age at the time of Bayley evaluation (X_i_β_3_), HOME scale (X_i_β_4_), sex (X_i_β_5_), maternal IQ (X_i_β_6_), breastfeeding (X_i_β_7_) and energy intake (X_i_β_8_)). In order to have a specific estimator for each Bayley Scale and every single covariable, the variable Bayley Scales_i_ are also interacted with each covariable. Z_ij_γ_i_ are random effects that included age at the time of Bayley evaluation and Bayley Scales for assessment j. The covariance between the two Bayley scales is included in the β calculations by specifying four covariance terms between the slope and the intercept of each scale and accounting for correlated error terms as well. Linear random slopes at age of evaluation were considered for all models. We used an unstructured variance-covariance matrix that allowed free estimation of all variances and covariances collapsing over age. See the supplement of [21] for the exact syntax used for the random part of the model.

Heteroscedasticity and linearity were graphically evaluated and no observations were excluded. Missing data were not imputed. A sensitivity analysis was perform using Bayley evaluations from 12 to 30 months of age (*n* = 244), since neurodevelopment assessment at early ages may be less robust and definitive [22]. In addition, we evaluated the possible interaction of DDE serum levels and PUFAs intake on child neurodevelopment. A probability value < 0.05 for the interaction was considered statistically significant. Statistical analysis was performed using the Stata ver.14.1 (StataCorp, College Station, TX, USA) statistical software package for Windows.

## Results

Participants in this study had significantly higher total breastfeeding than non-participants; in contrast, they had significantly lower HOME scale and maternal IQ (Table 1).

**Table 1:** Selected characteristics of participants and non-participants

Characteristics		Participants (*n*)	Non-participants (*n*)
Maternal			
Intellectual quotient	mean ± SD	87.50 ± 12.24 (274)	91.24 ± 13.24* (83)
Infant			
Breastfeeding (weeks)	%		
Never		7.25	7.95
≤ 12		23.91	36.36
> 12		68.84 (276)	55.68* (88)
Family			
HOME^a^	mean ± SD	30.37 ± 4.64 (264)	31.08 ± 4.63* (71)

^a^HOME Home Observation for Measurement of the Environment scale

*P-value< 0.05

The scores for MDI ranged from 86.24 to 98.41, while the PDI ranged from 86.81 to 97.63 (Table 2). Intakes of LA, ALA, and energy increased significantly from the first to the third trimester of pregnancy; while intakes of ARA, EPA and DHA, decreased (Table 3). Correlations for all PUFAs intake and DDE serum levels range from 0.005 to − 0.148 for LA during the third trimester and ARA during the first trimester; however, only five of this correlations resulted statistically significant (*p*-value < 0.05) (Data not shown). Maternal intakes of ALA and DPA during the first trimester of pregnancy were significantly and positively associated with MDI from 1 to 30 months of age. The above-mentioned associations remained significant after adjusting by maternal DDE serum levels (β_ALA-MDI_ = 0.63, 95% CI 0.25, 1.00; β_DPA-MDI_ = 0.10, 95% CI 0.02, 0.18) (Table 4). Sensitivity analysis showed that DPA and MDI association persisted positive and significant from 12 to 30 months of age (Additional file 1: Table S1). We found no interactions of DDE serum levels and PUFAs intake on child neurodevelopment (data not shown).

**Table 2:** Motor and mental development indices according to age at evaluation

Age (months)	(*n*)	Motor (PDI)	Mental (MDI)
		Mean ± SD	
1	(235)	97.63 ± 7.08	98.41 ± 4.30
3	(249)	86.81 ± 5.15	95.07 ± 5.36
6	(235)	95.03 ± 8.74	96.12 ± 4.20
12	(234)	90.54 ± 8.26	93.60 ± 7.32
18	(233)	92.24 ± 6.80	89.81 ± 7.78
24	(205)	95.58 ± 8.27	86.24 ± 10.66
30	(200)	93.16 ± 9.41	90.52 ± 8.27

**Table 3:** Polyunsaturated fatty acids and energy dietary intake during the first and third trimester of pregnancy

Dietary intake (daily)	Trimester	P50 (P10–P90)
Fatty acids		
Linoleic [LA] (g)	First	16.90 (7.40,32.39)
	Third	25.22 (17.06,36.12)*
Arachidonic [ARA] (mg)	First	93.31 (33.25,182.70)
	Third	88.23 (37.15,147.02)*
α-Linolenic [ALA] (g)	First	0.99 (0.46,2.94)
	Third	2.13 (1.49,3.23)*
Eicosapentaenoic [EPA] (mg)	First	9.86 (2.34,24.04)
	Third	8.96 (2.07,22.23)*
Docosapentaenoic [DPA] (mg)	First	4.47 (1.07,10.13)
	Third	4.23 (1.14,9.99)
Docosahexaenoic [DHA] (mg)	First	36.98 (9.68,77.71)
	Third	31.98 (9.14,66.96)*
Energy (Kilocalories)	First	1895.23 (1041.75,2882.10)
	Third	2619.11 (1759.91,3565.12)*

^*^P-value< 0.05; first trimester, *n* = 275; third trimester, *n* = 266

**Table 4:** Maternal polyunsaturated fatty acids and child neurodevelopment indices from 1 to 30 months of age

Polyunsaturated fatty acids	Mental (MDI)		Motor (PDI)	
	β (95% CI)^a^	β (95% CI)^b^	β (95% CI)^a^	β (95% CI)^b^
First trimester				
Linoleic (LA) g/day	0.04 (−0.01,0.08)	0.04 (− 0.01,0.09)	0.02 (− 0.04,0.07)	0.02 (− 0.04,0.07)
Arachidonic (ARA) mg/day	− 0.00 (− 0.01,0.00)	−0.00 (− 0.01,0.00)	−0.00 (− 0.01,0.00)	−0.00 (− 0.01,0.00)
Alfa linolenic (ALA) g/day	**0.62 (0.24,1.00)**	**0.63 (0.25,1.00)**	0.47 (0.01,0.93)	0.46 (0.00,0.93)
Eicosapentaenoic (EPA) mg/day	0.02 (−0.02,0.06)	0.02 (−0.02,0.06)	− 0.00 (− 0.05,0.04)	−0.00 (− 0.05,0.04)
Docosapentaenoic (DPA) mg/day	**0.10 (0.02,0.18)**	**0.10 (0.02,0.18)**	0.04 (−0.06,0.14)	0.04 (−0.06,0.14)
Docosahexaenoic (DHA) mg/day	0.01 (−0.01,0.02)	0.01 (−0.01,0.02)	− 0.00 (− 0.02, 0.01)	−0.00 (− 0.02,0.01)
Third trimester				
Linoleic (LA) g/day	0.03 (−0.05,0.11)	0.03 (−0.05,0.11)	− 0.01 (− 0.11,0.09)	−0.01 (− 0.11,0.09)
Arachidonic (ARA) mg/day	− 0.00 (− 0.01,0.01)	−0.00 (− 0.01,0.01)	0.00 (− 0.01,0.02)	0.00 (− 0.01,0.02)
Alfa linolenic (ALA) g/day	0.23 (− 0.42,0.89)	0.24 (− 0.42,0.89)	−0.01 (− 0.81,0.78)	−0.02 (− 0.81,0.78)
Eicosapentaenoic (EPA) mg/day	− 0.01 (− 0.04,0.03)	−0.01 (− 0.04,0.03)	−0.02 (− 0.06,0.02)	−0.02 (− 0.06,0.02)
Docosapentaenoic (DPA) mg/day	− 0.01 (− 0.09,0.07)	−0.01 (− 0.09,0.07)	−0.04 (− 0.14,0.05)	−0.05 (− 0.14,0.05)
Docosahexaenoic (DHA) mg/day	− 0.00 (− 0.02,0.01)	−0.00 (− 0.02,0.01)	−0.01 (− 0.02,0.01)	−0.01 (− 0.02,0.01)

^a^Adjusted by: child’s age at examination (months), Home Observation for Measurement of the Environment scale, gender, maternal intellectual quotient, breastfeeding (months) and energy intake (kilocalories).

^b^Previous model plus adjustment by maternal DDE serum levels (ng/g) during the first trimester of pregnancy.

Bold numbers correspond to statistically significant coefficients

## Discussion

Our results show that in a sample environmentally exposed to DDT, maternal ingestion of DPA during the first trimester of pregnancy is positively associated with MDI in children from 1 to 30 months. Likewise, our results suggest that dietary ALA may also be related to MDI.

We found no previous studies that reported a relationship between DPA and neurodevelopment of children younger than 30 months of age. However, in this same cohort, we identified a positive association between maternal DPA intake and the verbal component of the McCarthy scale in children from 42 to 60 months of age [23]. Additionally, in a clinical trial, where mothers were randomized to ingest oils sources of DPA from 18 weeks of gestation to 3 months after delivery, plasma DPA, measured among their children at 4 weeks of age, was positively correlated with children mental development at 4 years of age [24]. DPA might benefit child neurodevelopment since it is a precursor of resolvins and neuroprotectins [25]; however, more studies are required to confirm the aforementioned findings.

Regarding ALA maternal intake, previous studies did not find association between dietary maternal ALA intake and child neurodevelopment at 6 months [26], 2 and 3 years of age [27, 28]. In the same way, we did not observe an association between maternal dietary ALA and child neurodevelopment from 12 to 30 months of age; however, when we included child neurodevelopment evaluations at 1, 3 and 6 months of age, positive significant associations emerged. This finding should be interpreted with caution considering that early neurodevelopment assessments have lower predictive value than latter ones [22].

Inconclusive information is available regarding child neurodevelopment and DHA [5, 29], which plays an important role on signal transduction, neurotransmission, and neurogenesis. It has been suggested that lack of consideration of polymorphisms, as well as the presence of heterogeneity among neurodevelopment assessment methods, in addition to the variations in timing, type, duration and concentration of supplementation of PUFAs may determine the inconsistencies among studies [30]. In previous clinical trials, where DHA was associated with at least one component of child neurodevelopment, maternal supplementation of DHA ranged from 200 to 2200 mg/day [5, 29]. This may explain the lack of association between child neurodevelopment and DHA in this study where median maternal intake of DHA was much lower (about 35 mg/day).

Previous cohorts studies have negatively linked ARA intake during pregnancy and child neurodevelopment [27, 31]. We did not find a relationship between ARA and child neurodevelopment, probably due to the small sample size. ARA plays a role in cell signaling and synaptic transmission via specific eicosanoids and leukotrienes [7], but also produces contrasting inflammatory effects [32]. Additionally, although we do not have reproducibility coefficients for each PUFA, the reproducibility coefficient for total intake of PUFAs in our FFQ was 0.38. Its validity against an average of 16 24-h recall questionnaires range from 0.12 to 0.21, which we may use as an approximation of the magnitude of this instrument’s measurement error [16]. As a result, non-differential measurement error may have underestimated the associations of interest. In this context, we cannot rule out that ARA and DHA consumption is associated with child neurodevelopment.

To our knowledge, this is the first study that asses maternal intake of PUFAs in a sample environmentally exposed to DDT. After adjusting for DDE, the association between DPA and MDI was similar, which suggests that even in the presence of this neurotoxicant, DPA improve the MDI. However, the lack of information about maternal intake of PUFAs and child neurodevelopment in other samples exposed to this neurotoxicant prevented comparison with other studies.

We previously reported that DDE serum levels and intake of PUFAs interact on child neurodevelopment in children from 42 to 60 months of age in this same cohort, where neurodevelopment was measured using the McCarthy scales [23]. In the present report, no interaction between DDE serum levels and intake of PUFAs was found, a result that may be partially explained by the low predictive validity of neurodevelopmental assessments at early ages [22]. Lack of precise measurements of intake of PUFAs may also be a factor in failure to find the above-mentioned interactions at early developmental ages.

To reduce the possibility of type I error we combined all Bayley indexes in a single model. While participant children received less stimulation at home and their mothers had lower maternal intellectual quotient they received longer duration of lactation; thus, it is difficult to know the impact that these differences may have had on the PUFAs and child neurodevelopment relationship, which may have reduced the external validity of our results. No information on children intake of PUFAs was available in this analysis; however, most previous studies suggest a lack of association of child intake of PUFAs with child neurodevelopment [33].

Reduced study power as a result of the small sample size limited our ability to detect small effects as statistically significant. We should view the results presented here as preliminary until larger samples of subjects are studied.

People living in malarial countries where the use of DDT indoor residential spray is currently performed, may have DDE serum levels between 200 to 77,900 ng/g, that are lower than those for occupational exposure (7100–131,800 ng/g) [34]. DDE serum levels in our study population ranged from 69.44 to 28,410.42 ng/g, which might suggest contemporaneous environmental exposure to DDT during the pregnancy phase of the study.

## Conclusion

DPA may benefit neurodevelopment even in populations environmentally exposed to DDT. Our results strengthen the importance of intake of PUFAs during the prenatal period.

## Additional file

Additional file 1: **Table S1.** Maternal polyunsaturated fatty acids and child neurodevelopment indices from 12 to 30 months. (DOCX 20 kb)

## Abbreviations

ALA: α-linolenic acid
ARA: Arachidonic acid
DDE: 1,1-dichloro-2,2-bis(p-chlorophenyl) ethylene
DDT: 1,1,1-trichloro-2,2-bis(p-chlorophenyl)ethane
DHA: Docosahexaenoic acid
DPA: Docosapentaenoic acid
EPA: Eicosapentaenoic acid
LA: Linoleic acid
MDI: Mental development index
PDI: Psychomotor development index
PUFAs: Polyunsaturated fatty acids

## References

[1] Fleith M, Clandinin MT (2005). Dietary PUFA for preterm and term infants: review of clinical studies. Crit Rev Food Sci Nutr.

[2] Hibbeln JR, Gow RV (2014). Omega-3 fatty acid and nutrient deficits in adverse neurodevelopment and childhood behaviors. Child Adolesc Psychiatr Clin N Am.

[3] Benatti P, Peluso G, Nicolai R, Calvani M (2004). Polyunsaturated fatty acids : biochemical , nutritional and epigenetic properties. J Am Coll Nutr.

[4] Crupi R, Marino A, Cuzzocrea S (2013). N-3 fatty acids: role in neurogenesis and neuroplasticity. Curr Med Chem.

[5] Campoy C, Escolano-Margarit V, Anjos T, Szajewska H, Uauy R (2012). Omega 3 fatty acids on child growth, visual acuity and neurodevelopment. Br J Nutr.

[6] González HF, Visentin S (2016). Nutrients and neurodevelopment: lipids. Update. Arch Argent Pediatr.

[7] Strain JJ, Davidson PW, Bonham MP, Duffy EM, Stokes- A, Thurston SW (2008). Associations of maternal long chain polyunsaturated fatty acids, methyl mercury, and infant development in the Seychelles child development nutrition study. Neurotoxicology.

[8] Torres-Sánchez L, Rothenberg SJ, Schnaas L, Cebrián ME, Osorio E, Hernández MDC (2007). In utero p,p’-DDE exposure and infant neurodevelopment: a perinatal cohort in Mexico. Environ Health Perspect.

[9] Rice D, Barone S (2000). Critical periods of vulnerability for the developing nervous system: evidence from humans and animal models. Environ Health Perspect.

[10] Wolff MS, Anderson Ha, Britton J a, Rothman N (2007). Pharmacokinetic variability and modern epidemiology - the example of dichlorodiphenyltrichloroethane, body mass index, and birth cohort. Cancer Epidemiol Biomark Prev.

[11] Channon KE, Méndez-Galván JF, Galindo-Jaramillo JM, Olguín-Bernal H, Borja-Aburto VH (2003). Cooperative actions to achieve malaria control without the use of DDT. Int J Hyg Environ Health.

[12] Landrigan PJ, Fuller R, Acosta NJR, Adeyi O, Arnold R, Basu N (2018). The lancet commission on pollution and health. Lancet.

[13] Torres-Sánchez L, Schnaas L, Cebrián ME, Hernández MDC, Valencia EO, García Hernández RM (2009). Prenatal dichlorodiphenyldichloroethylene (DDE) exposure and neurodevelopment: a follow-up from 12 to 30 months of age. Neurotoxicology.

[14] Bayley N. Bayley Scales of Infant Development. Psychol Corp. 1993;2nd ed.

[15] Gomaa A, Hu H, Bellinger D, Schwartz J, Tsaih S-W, Gonzalez-Cossio T (2002). Maternal bone Lead as an independent risk factor for fetal neurotoxicity: a prospective study. Pediatrics.

[16] Hernández-Avila M, Romieu I, Parra S, Hernández-Avila J, Madrigal H, Willett W (1998). Validity and reproducibility of a food frequency questionnaire to assess dietary intake of women living in Mexico City. Salud Publica Mex.

[17] USDA. Agricultural Research Service. USDA National Nutrient Database for Standard Reference, Release 20. USDA. Nutrient data laboratory home page. 2007.

[18] Willett WC, Howe GR, Kushi L (1997). Adjustmentfor total energy intake in epidemiologic studies. Am J Clin Nutr.

[19] Wechsler D. WAIS-Español. Escala de inteligencia para Adultos. Man Mod SA. 1981;Manual.

[20] Totsika V, Sylva K (2004). The home observation for measurement of the environment revisited. Child Adolesc Ment Health.

[21] Baldwin SA, Imel ZE, Braithwaite SR, Atkins DC (2014). Analyzing multiple outcomes in clinical research using multivariate multilevel models. J Consult Clin Psychol.

[22] Dietrich KN, Eskenazi B, Schantz S, Yolton K, Rauh V a, Johnson CB (2005). Principles and practices of neurodevelopmental assessment in children: lessons learned from the centers for Children’s environmental health and disease prevention research. Environ Health Perspect.

[23] Ogaz-Gonzalez R, Merida-Ortega A, Torres-Sanchez L, Schnaas L, Hernandez-Alcaraz C, Cebrian M (2018). Maternal dietary intake of polyunsaturated fatty acids modifies association between prenatal DDT exposure and child neurodevelopment: a cohort study. Environ Pollut.

[24] Helland IB, Smith L, Saarem K, Saugstad OD, Drevon C (2003). a. Maternal supplementation with very-long-chain n-3 fatty acids during pregnancy and lactation augments Children’s IQ at 4 years of age. Pediatrics.

[25] Kaur G, Cameron-Smith D, Garg M, Sinclair AJ (2011). Docosapentaenoic acid (22,5n-3): a review of its biological effects. Prog Lipid Res.

[26] Kim H, Kim H, Lee E, Kim Y, Ha EH, Chang N. Association between maternal intake of n-6 to n-3 fatty acid ratio during pregnancy and infant neurodevelopment at 6 months of age: results of the MOCEH cohort study. Nutr J. 2017;16(1):–10. 10.1186/s12937-017-0242-9 PMC5395920 28420388

[27] Bernard JY, De AM, Forhan A, De L-g B, Charles M, Heude B, et al. The dietary n6 : n3 fatty acid ratio during pregnancy is inversely associated with child neurodevelopment in the EDEN mother-child cohort. J Nutr. 2013:1481–8. 10.3945/jn.113.178640 23902952

[28] Bernard JY, Armand M, Garcia C, Forhan A, De Agostini M, Charles MA (2015). The association between linoleic acid levels in colostrum and child cognition at 2 and 3 y in the EDEN cohort. Pediatr Res.

[29] Gould J, Smithers L, Makrides M (2013). The effect of maternal omega-3 (n-3) LCPUFA supplementation during pregnancy on early childhood cognitive and visual development: a systematic review and meta-analysis of randomized controlled trials. Am J Clin Nutr.

[30] Meldrum SJ, Smith MA, Prescott SL, Hird K, Simmer K (2011). Achieving definitive results in long-chain polyunsaturated fatty acid supplementation trials of term infants: factors for consideration. Nutr Rev.

[31] Strain JJ, Davidson PW, Thurston SW, Harrington D, Mulhern MS, McAfee a J (2012). Maternal PUFA status but not prenatal methylmercury exposure is associated with Children’s language functions at age five years in the Seychelles. J Nutr.

[32] Luchtman DW, Song C. Cognitive enhancement by omega-3 fatty acids from child-hood to old age: findings from animal and clinical studies. Neuropharmacology. 2012:1–16. 10.1016/j.neuropharm.2012.07.019 22841917

[33] Meldrum S, Simmer K (2016). Docosahexaenoic acid and neurodevelopmental outcomes of term infants. Ann Nutr Metab.

[34] World Health Organisation. Environmental Health Criteria 241 DDT in door residual spraying: human health aspects. 2011.

