# Supplementary material for: Polyunsaturated fatty acids and child neurodevelopment among a population exposed to DDT: a cohort study
Source: Environ Health. 2019 Feb 28;18:17. doi: 10.1186/s12940-019-0456-8 (PMC6396452; doi:10.1186/s12940-019-0456-8)
Supplement: Supplementary file 1 — Table S1. Maternal polyunsaturated fatty acids and child neurodevelopment indices from 12 to 30 months. (DOCX 20 kb) [file 12940_2019_456_MOESM1_ESM.docx]

| **Additional file 1: Table S1.** Maternal polyunsaturated fatty acids and child neurodevelopment indices from 12 to 30 months | | | | |
| --- | --- | --- | --- | --- |
| **Polyunsaturated fatty acids** | **Mental (MDI)** | | **Motor (PDI)** | |
| **First trimester** | β (95% CI)ᵅ | β (95% CI)ᵇ | β (95% CI)ᵅ | β (95% CI)ᵇ |
| Linoleic (LA) g/day | 0.03 (-0.05,0.11) | 0.03 (-0.05,0.11) | -0.00 (-0.08,0.07) | -0.00 (-0.08,0.07) |
| Arachidonic (ARA) mg/day | -0.00 (-0.01,0.01) | -0.00 (-0.01,0.01) | -0.00 (-0.01,0.01) | -0.00 (-0.01,0.01) |
| Alfa linolenic (ALA) g/day | 0.52 (-0.14,1.18) | 0.54 (-0.12,1.21) | 0.25 (-0.34,0.0.85) | 0.25 (-0.35,0.85) |
| Eicosapentaenoic (EPA) mg/day | 0.03 (-0.04,0.09) | 0.03 (-0.04,0.09) | -0.02 (-0.08,0.04) | -0.02 (-0.08,0.04) |
| Docosapentaenoic (DPA) mg/day | **0.20 (0.05,0.34)** | **0.19 (0.05,0.34)** | 0.06 (-0.07,0.20) | 0.06 (-0.07,0.20) |
| Docosahexaenoic (DHA) mg/day | 0.01 (-0.01,0.03) | 0.01 (-0.01,0.03) | -0.01 (-0.03, 0.02) | -0.01 (-0.03,0.02) |
| **Third trimester** |  |  |  |  |
| Linoleic (LA) g/day | 0.09 (-0.05,0.23) | 0.09 (-0.05,0.24) | -0.01 (-0.14,0.12) | -0.01 (-0.14,0.12) |
| Arachidonic (ARA) mg/day | -0.01 (-0.02,0.01) | -0.01 (-0.02,0.01) | -0.00 (-0.02,0.01) | -0.00 (-0.02,0.01) |
| Alfa linolenic (ALA) g/day | 0.75 (-0.39,1.89) | 0.75 (-0.39,1.90) | 0.18 (-0.86,1.23) | 0.18 (-0.87,1.23) |
| Eicosapentaenoic (EPA) mg/day | -0.02 (-0.08,0.04) | -0.02 (-0.07,0.04) | -0.03 (-0.09,0.02) | -0.03 (-0.09,0.02) |
| Docosapentaenoic (DPA) mg/day | -0.05 (-0.19,0.10) | -0.04 (-0.19,0.10) | -0.08 (-0.21,0.05) | -0.08 (-0.21,0.05) |
| Docosahexaenoic (DHA) mg/day | -0.01 (-0.03,0.01) | -0.01 (-0.03,0.01) | -0.01 (-0.03,0.01) | -0.01 (-0.03,0.01) |
| ᵅ Adjusted by: child’s age at examination (months), Home Observation for Measurement of the Environment scale, gender, maternal intellectual quotient, breastfeeding (months) and energy intake (kilocalories). ᵇ Previous model plus adjustment by maternal DDE serum levels (ng/g) during the first trimester of pregnancy.  Bold numbers correspond to statistically significant coefficients. | | | | |
